# Gutta Percha—Its Uses, &c.

**Published:** 1848-04

**Authors:** G. F. J. Colburn

**Affiliations:** Dentist, Newark, N. J.


### ARTICLE VI.

*Gutta Percha—Its Uses, &c.* By G. F. J. COLBURN, Dentist, Newark, N. J.

THE new substance, similar to caoutchouc, recently introduced to the notice of the public, called gutta percha, is the coagulated sap and milky juice, of a whitish grey color, which exudes from a tree belonging to the natural order, *sapotaceac*, a native of the island of Singapore, and the dense forests of the Malayan peninsula. It has many curious properties. Below the temperature of fifty degrees, it is of the consistency and hardness of wood. Unlike caoutchouc, it has little elasticity, possessing much tenacity when drawn out, and remains without contracting.

This material may be applied to various useful purposes. For the surgeon it makes excellent bougies, catheters, enema pipes, splints, &c.

The dentist may find in it a substitute for wax for taking impressions of the mouth, it will not adhere to the teeth, and becomes so hard in a few moments as to be readily removed, without danger of being disturbed by coming in contact with the cheeks and angles of the mouth. Also, if, on withdrawing the plaster cast, any of the teeth remain in the mould, the pieces can be removed without injury to the impression as would be the case with wax.

I have tried frequent experiments with this material, and succeeded admirably in obtaining correct impressions. When used for this purpose, the gutta should be thoroughly soaked in boiling water, then kneaded and moulded in the same way as wax to fit the case; both should then be immersed, and the water shaken off, and immediately placed in the mouth, and firmly pressed to its place.

The operator can greatly facilitate the hardening of the gutta, by dipping his finger in cold water, and bathing the case. It should be allowed to remain in the mouth for two or three minutes, when it will be sufficiently congealed to be removed with safety. The same piece can be remoulded any number of times, and is by no means unpleasant to the patient while in the mouth.

I have, also, instituted a number of experiments with this substance in order to test its practicability for filling teeth; the utility of which time must determine.

As this article has been but a short time known to the public, and possesses many properties that may be turned to the use of the dentist, is it not deserving the attention of the profession?

